# Effects of lumbar discectomy on disability and depression in patients with chronic low back pain

**DOI:** 10.5812/kowsar.22287523.1529

**Published:** 2011-07-01

**Authors:** Gholamreza Farzanegan, Mohsen Alghasi, Saeid Safari, Seyyed Ali Ahmadi

**Affiliations:** 1 Department of Neurosurgery, Baqiyatallah University of Medical Sciences, Tehran, IR Iran; 2 Department of Neurosurgery, Tehran University of Medical Sciences, Tehran, IR Iran; 3 Department of Anesthesiology, Tehran University of Medical Sciences, Tehran, IR Iran

**Keywords:** Depression, Discectomy, Low Back Pain, Sciatica, Herniated Disc

## Abstract

**Background:**

Back pain is one of the most prevalent health problems for which physicians are consulted. Back pain has many economic impacts, such as sickness absences and long-term disability. The prevalence of major depression in patients with chronic low back pain is approximately 3 to 4 times greater than the prevalence rate reported in the general population.

**Objectives:**

This study was designed to evaluate the depression and disability improvement after lumbar discectomy compared with presurgery levels in patients with chronic low back pain and radicular leg pain.

**Patients and Methods:**

One hundred forty-eight patients with chronic low back pain and radicular leg pain were included in this analytic observational study. The study evaluated several main variables, including age; sex; educational level; job; height; weight; and patient history of abortion, leg pain, back pain, smoking, trauma, number of previous pregnancies, driving, long-term sitting, lifting heavy bodies, and disability and depression before and 6 and 12 months after laminectomy.

**Results:**

The depression and disability scores of patients before lumbar discectomy significantly decreased after surgery.

**Conclusions:**

Our results indicate that lumbar discectomy surgery significantly improved depression and disability in patients with chronic herniated discs.

## 1. Background

Back pain is one of the most common health problems for patients and physicians (1). Back pain has many economic impacts, such as sickness absences and long-term disability (2). In the past, the advice to patients with low back pain usually was to stay active because very few interventions could ease back pain in the acute stage (3). The recommendation to stay active was also made for patients in the subacute stages of back pain (4, 5). In line with these findings, clinical guidelines have developed to change clinical practice (6, 7). To prevent disability, patients are advised not to rest when experiencing back pain, but to stay active (8). Back pain is also related to mental health. The literature has demonstrated a relationship between chronic pain and depression (9, 10). It has been reported that between 50 and 65 percent of chronic pain patients also have a diagnosis of depression (11). The relationship is complex and multifactorial, including a lower tolerance for pain in people with depression (12). Additionally, an avoidance of activities that may be directly or indirectly associated with the effectiveness of the therapeutic process (13). Advances in physical therapy and general opinion about disability should bring about a change in the incidence of disability. Therefore, advances in the management of back pain should be demonstrable in changing incidence of disability of back pain. Partial or total laminectomy is one of the surgical approaches for patients with chronic low back pain.

## 2. Objectives

The present study was designed to evaluate disability and depression improvement after lumbar discectomy in patients with chronic low back pain.

## 3. Patients and Methods

One hundred forty-eight patients with chronic low back pain (3 months after disease onset) were included in the study. This was an analytic observational study that was conducted with cross sectional methods.

### 3.1. Study samples and variables

The study sample included 148 patients with chronic low back pain who were referred for surgery to Baghiatollah Hospital from January 2009 to May 2010. This study evaluated several main variables, including age; sex; educational level; job; height; weight; and patient history of abortion, leg pain, back pain, smoking, trauma, pregnancy, and number of that, driving, long-term sitting, lifting heavy bodies, and disability and depression before and 6 and 12 months after laminectomy. Disability was assessed by Rolland and Morris’s questionnaire. The questionnaire was used to evaluate disability in patients’ previous reports (14, 15). Depression was measured by the Beck Depression Inventory, which includes 21 questions with multiple-choice answers. Written informed consent was obtained from all patients. All variables were recorded by questionnaire.

### 3.2. Statistical analysis

All data were entered into the computer via SPSS software Version 14.0 (SPSS Inc, Chicago Ill). The aim of the present study was to evaluate the influence of lumbar discectomy on the improvement of disability and depression in patients with chronic low back pain. One sample t-tests were used to compare disability- and depression-score improvement with the lowest disability- and depression-score improvement (no changes over the time in disability situation) before and after lumbar discectomy. Disability- and depression-score improvement (difference between disability score at the beginning and end points) were also compared by sex, educational level (less than a high school diploma versus diploma or higher) and body mass index (BMI; under 25 and above), with independent-sample t-tests. A two-tailed significance level of 0.05 was used to detect significant differences between variables.

## 4. Results

Seventy male (46.6%) and 78 (53.4%) female patients participated in the study. Fifty-six (38.1%) patients had a military job, and 77 patients (53.4%) had a diploma or higher educational level. The mean age of the study patients was 44.33 ± 11.53 years. The mean height and weight were 165.52 ± 11.17 cm and 75.21 ± 11.47 kg, respectively. The mean BMI was 27.62 ± 4.39 (Table 1). In the analysis of the patient histories, 106 patients (72.1%) had a history of lifting heavy bodies, 101 patients (68.7%) had a history of long-term sitting, 59 patients (40.1%) had a history of driving, 20 patients (13.6%) had a history of smoking, 75 patients (51%) had a history of pregnancy, and 22 patients (15%) had a history of abortion (Table 2). 

**Table 1.** Demographic variables in patients with chronic low back pain

**Qualitive variables [No. (%)]**	
Male	70 (46.6)
Diploma or higher	77 (53.4)
Have a military Job	56 (38.1)
**Quantities variables (Mean ± SD)**	
Age (y)	44.33 ± 11.53
Height (cm)	165.52 ± 11.17
Weight (kg)	75.21 ± 11.47
BMI a (kg/m2)	27.62 ± 4.39

**Table 2.** History of some activities before laminectomy surgery in our study samples

Activitiy	No. (%)
**Lifting**	106 (72.1)
**Long-term sitting **	101 (68.7)
**Driving **	59 (40.1)
**Smoking**	20 (13.6)
**Pregnancy**	75 (51)
**Abortion**	22 (15)

### 4.1. Disability score improvement

In comparing disability-score improvement in patients with chronic low back pain with the lowest disability-score improvement, the mean disability-score improvement at 6 months (58.21 ± 21.28; p =0.0) and 12 months (74.69 ± 28.12; p=0.0) after lumbar discectomy was significantly different from the lowest disability-score improvement.

### 4.2. Depression score improvement

In comparing the Beck depression scores of patients with chronic low back pain at baseline and 6 and 12 months after lumbar discectomy, depression status had improved significantly by 6 and 12 months after lumbar discectomy (p = 0.0) (Table 3). 

**Table 3.** Depression status in our samples before and after lumbar discectomy

Depression status	Number	Frequency	P value
**Depression status at baseline**			
**Not depressed (0-9) (y)**	45	30.6	
**Mildly depressed (10-19) (y)**	45	30.6	
**Moderately depressed (20-29) (y)**	39	26.5	
**Severely Depressed (30-39) (y)**	12	8.2	
**Very severely depressed ( 40 ≤ ) (y)**	6	4.1	
**Depression status 6 months after evaluation**			0.0^a^
**Not depressed (0-9) (y)**	74	50.3	
**Mildly depressed (10-19) (y)**	51	34.7	
**Moderately depressed (20-29) (y)**	14	9.5	
**Severely Depressed (30-39) (y)**	7	4.8	
**Very severely depressed ( 40 ≤ ) (y)**	1	0.7	
**Depression status 12 months after evaluation**			0.0^b^
**Not depressed (0-9) (y)**	82	55.8	
**Mildly depressed (10-19) (y)**	43	29.3	
**Moderately depressed (20-29) (y)**	14	9.5	
**Severely Depressed (30-39) (y)**	7	4.8	
**Very severely depressed ( 40 ≤ ) (y)**	1	0.7	

^a^ This P value compared the baseline and 6 months after lumbar discectomy.

^b^ This P value compared the baseline and 12 months after lumbar discectomy.

### 4.3. Comparison of depression and disability improvement in patients with chronic low back pain by gender

Female patients had a significantly higher mean of depression improvement than male patients (9.26 ± 13.92 vs. 2.70 ± 12.70; p = 0.003). This difference wasn’t significant in mean of disability improvement (14.66 ± 6.25 vs. 14.48 ± 5.98; p = 0.85).

### 4.4. Comparison of depression and disability improvement in patients with chronic low back pain by educational level

The mean of disability improvement was not significantly different between the two educational groups (less than a high school diploma and diploma or higher; 6.46 ± 14.34 vs. 5.84 ± 13.10; p = 0.79). Mean of disability improvement hadn’t significant difference between two (under and higher than diploma) (14.24 ± 6.24 vs. 14.87 ± 5.99; p = 0.54).

### 4.5. Comparison of depression and disability improvement in patients with chronic low back pain by BMI

The mean of disability improvement was not significantly different between the two BMI groups (under 25 vs. above; 8.05 ± 13.97 vs. 5.32 ± 13.58; p = 0.21). The mean of disability improvement was not significantly different between the two BMI groups (13.93 ± 6.46 vs. 14.84 ± 5.95; p = 0.41).

## 5. Discussion

In our evaluation of disability and depression improvement in patients with chronic low back pain, both disability and depression significantly improved 6 and 12 months after lumbar discectomy surgery. Female patients experienced a significantly higher mean of depression improvement than male patients. Katz et al. reported that patients with functional disabilities are significantly less satisfied with the results of surgery for herniated discs (16). Cavusoglu et al. reported that for spinal surgery, unilateral laminectomy approaches allowed sufficient and safe decompression of the neural structures and adequate preservation of vertebral stability and resulted in a highly significant reduction of symptoms and disability (17). In contrast to the present findings, Graver et al. reported that female sex was significantly related to lower frequencies of return to work in patients with herniated discs (18). Loupasis et al. reported that female gender was a predisposing factor for unsatisfactory outcomes in herniated-disc surgery (19). Contrary to our results, Ljunggren et al. found that BMI and long duration of absence due to illness were significantly related to poor outcomes in herniated-disc surgery (18). The prevalence of major depression in patients with chronic low back pain is approximately 3 to 4 times greater than the prevalence reported in the general population (20). Some researchers have evaluated the relationship between the perception of pain and psychological distress after treatment of low back pain. Their results suggest that the strength of the relationship between chronic pain perception and distress is related to both aspects of the patient's personality and characteristics of their illness and, interestingly, not to the duration of their pain (21). Practitioners who focus on treating somatic structures, such as chiropractors, osteopaths, and physiotherapists, may tend to minimize the importance of these psychological factors in the promotion of pain management (22). Depression associated with low back pain and other types of pain is often different from the classical signs and symptoms of clinical depression (23). In particular, much of the emotional distress in patients with chronic pain does not include the common cognitive characteristics associated with clinical depression, such as feelings of shame, guilt, anxiety, and anger. This is despite the fact that patients are often hostile toward their medical practitioners for not resolving their low back pain (24). Researchers suggested that, instead of searching for a direct causal path, we must accept that affect and sensory information are processed in parallel, and even if one of the processing channels is more dominant, the relationship is most likely cyclical. They conclude that medical practitioners should focus on who is more vulnerable to negative affect and stress as that may allow them to help patients more effectively (23). Banks and Kerns reported that "there is growing empirical evidence to suggest that depression is most commonly secondary to chronic pain" (25). Screening with a depression-specific tool such as the Beck Depression Inventory may be appropriate in instances of high suspicion of an underlying depressive state. This and other questionnaires are frequently used to identify the disability associated with the depression rather than the psychosocial factors associated with the depression. Therefore, care must be taken in the use of these scales(26). The present study has some limitations. First, we detected the disability and depression score in our samples with the Rolland Morris and Beck questionnaires. Other methods might show different results. Second, this study was restricted to one local Iranian population. Third, this study was conducted in a single center, and therefore the results cannot be generalized to populations in other countries or centers in Iran. Despite these limitations, our results indicate that lumbar discectomy surgery significantly improved the disability and depression of patients with chronic herniated discs.
